# Anti-TPO Antibodies Diffusion through the Placental Barrier during Pregnancy

**DOI:** 10.1371/journal.pone.0084647

**Published:** 2014-01-31

**Authors:** Jérémy Seror, Gaëlle Amand, Jean Guibourdenche, Pierre-François Ceccaldi, Dominique Luton

**Affiliations:** 1 Paris Diderot, Université Paris VII, Paris, France; 2 Department of Gynecology and Obstetrics, Beaujon-Bichat Hospital, AP-HP, Clichy, France; 3 Department of Hormonal and Metabolic Biology, Cochin Hospital, AP-HP, Paris, France; University of Michigan Medical School, United States of America

## Abstract

**Background:**

Hashimoto’s thyroiditis is the principal aetiology of hypothyroidism with presence of anti-thyroperoxidase antibodies (anti-TPO). The association between anti-TPO and foeto-placental complications has been observed in previous studies. To go further in the understanding, the current study compares the level of anti-TPO in maternal blood and in the cord blood of her fetus at the moment of childbirth to demonstrate the passage of anti-TPO through the placenta barrier.

**Methods and Findings:**

This study was realised in a maternity ward located in the Northern district of Paris, France from 2006 to 2007. Women with normal pregnancy were included in a first study and only women with no abnormal thyroid dosage at baseline and tested positive with anti-TPO were prospectively enrolled. Maternal blood samples were collected in the third trimester and at the arrival to the ward when patients came to deliver. After delivery, cord blood sample was collected. Pearson’s correlation coefficient was computed. 5941 patients delivered in the ward during the study, 33 pregnant women were included. We found a correlation between the anti-TPO levels in maternal and in the cord blood of their fetus with a correlation coefficient of 0.98 and a p-value<0.001.

**Conclusions:**

This is the first demonstration of the free passage through the placental barrier of anti-TPO from the mother to the fetus at the moment of childbirth. These findings can be extrapolated all along pregnancy and open the door to a direct action of the anti-TPO on fetus and to a possible action on the fetal thyroid.

## Introduction

Hypothyroidism is one of the most common endocrine disorders [1]. Prevalence of hypothyroidism during pregnancy is from 1% to 2% [2]–[4]. Autoimmune disease is the principal etiology of hypothyroidism and particularly Hashimoto's thyroiditis with anti-thyroperoxidase antibodies (anti-TPO) [5]–[7]. Hypothyroidism during pregnancy increases the risk to develop vasculo-placental complications [8], [9] such as gravidic hypertension, pre-eclampsia, preterm, abruption of placenta and post partum haemorrhage or post partum thyroiditis [10], [11]. Fetal complications described are lower fetal birth weight and fetal distress [12]. However, the association between presence of anti-TPO, and such foeto-maternal complications have been observed [13], [14] and some authors in literature believe that anti-TPO could play a role in vasculo-placental complications occurrence [15]. Otherwise, although maternal thyroid hormones do not pass easily placental barrier, they are essential for the formation of the nervous system from the beginning of gestation [16] as maternal iode transfer. The balance of thyroid hormones is measured by the level of free triiodothyronine (FT3), free thyroxine (FT4) and thyreo stimulating hormone (TSH) during pregnancy [17]. To go further in the understanding, the current study compares the level of anti-TPO in maternal blood to the level of anti-TPO in cord blood of her fetus at the moment of childbirth to demonstrate the passage of anti-TPO through the placenta barrier, and compares anti-TPO levels and TSH, FT4, FT3 values from the mother and her fetus at the same time.

## Materials and Methods

**Figure 1:**
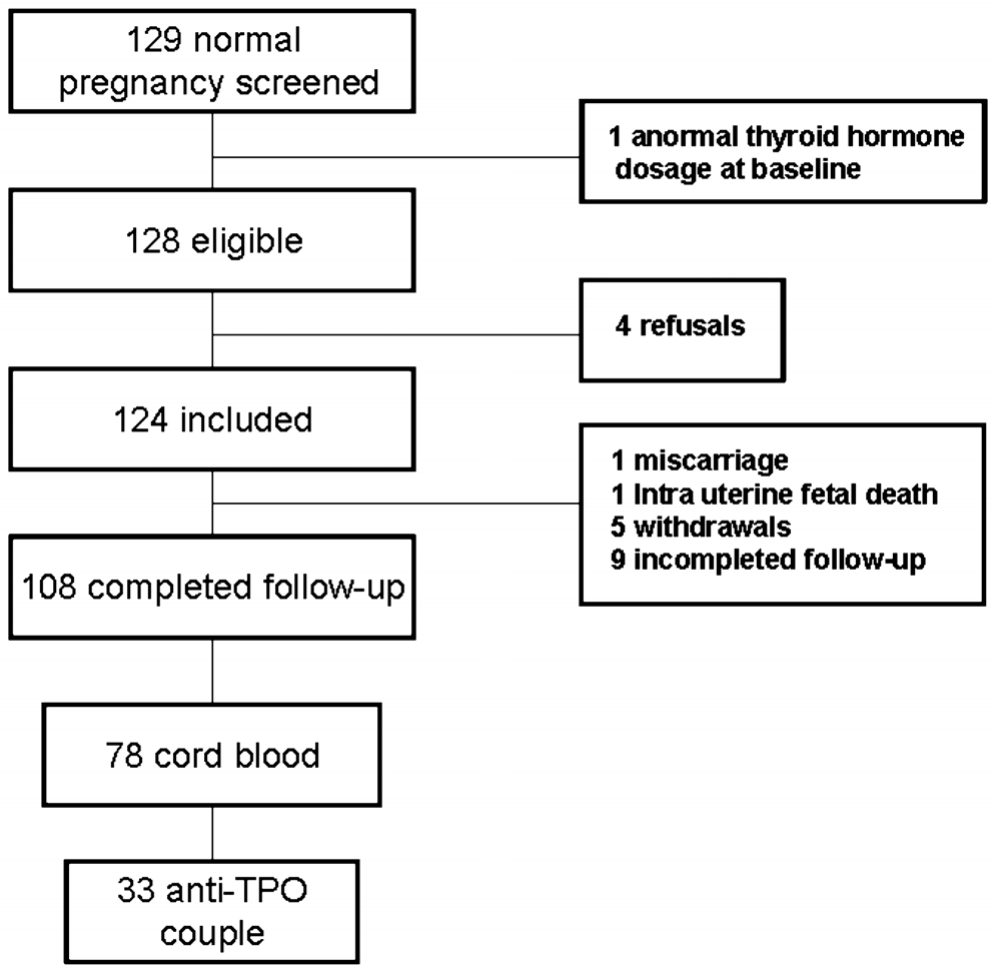
Chart-Flow.

This study is part of a first study [18] realised from 2006 to 2007 in the maternity ward of the pediatric hospital of Robert Debré Hospital located in the northern district of Paris, France. The study planned to prospectively recruit follow-up, maternal blood samples and cord blood samples of 110 pregnant patients receiving routine prenatal care. Inclusion criteria were normal pregnancy, having signed the study informed consent document, and being covered by the French public healthcare insurance system. The appropriate ethics committee (Comité de protection des personnes d’Ile de France) approved the study protocol may 12, 2005, under the number 0511132. Exclusion criteria were the presence of chronic disease; iodine supplementation; current or past thyroid disease; fetal abnormalities; multiple pregnancy; pregnancy induced using assisted reproductive technology; and abnormal thyroid hormone concentrations at baseline. Of 129 patients who were invited to participate in the study, 4 refused participation and 1 had abnormal serum thyroid hormone concentrations. Of the remaining 124 patients, 108 (87%) attended all the study visits. One patient had a miscarriage and another fetus died *in utero* at 22 weeks of gestational. Five additional patients asked to leave the study later during the pregnancy, usually at the request of the husband. All Pregnancy follow-up were realized in the ward and were seen monthly by an obstetrician. None of the study patients delivered prematurely. Cord blood samples were obtained at delivery from 72 fetuses from 72 pregnancies. Among these 72 pregnancies, were selected only women with no abnormal thyroid dosage at baseline tested positive with anti-TPO (Figure 1).

Maternal blood samples were collected in the third trimester and at the arrival to the ward when patients came to deliver. After delivery, cord blood sample was collected. All blood samples were tested after collection of all study data was complete. All sera were frozen at −80°C until use. Serum FT3, FT4, and TSH were assayed on an ACS-180 SE automate using chemiluminescence immunoassay. Variability was 3.3%, for FT3, 6.6%, for FT4, and 8.4% for TSH. The limits of detection were 0.3 pm/L, 1.3 pml/L, and 0.02 IU/L, respectively.

Serum levels of anti-TPO in maternal and cord blood were detected by direct chemiluminescence immunoassay on an ACS-180SE automate. The limit of detection was 15 UI/L.

Results are expressed as numbers and/or percentages for categorical variables and means ± standard deviation (SD), or median [min; max], for continuous variables, unless stated otherwise.

We compared hormonal levels between mother blood samples at the third trimester of gestation and fetal cord blood samples of their fetus. Each pregnancy was considered as an independent event. All quantitative variables were compared with non parametric Wilcoxon’s test. Pearson’s correlation coefficient was computed to assess the link between maternal and fetal thyroid parameters (FT3, FT4, TSH, and anti-TPO). All tests were two-tailed, p-values less than 0.05 were considered significant. Statistical analyses were performed using Statview Software V5 (SAS Institute Inc, Chicago, IL, USA; Copyright 1992–1998 Version 5.0.).

## Results

During the period of the study, a total of 5941 patients delivered in the institution. The 33 couples of blood samples from mothers with positive anti-TPO and cord blood samples of their fetus were matched for analyse in this study (Figure 1).

**Figure 2:**
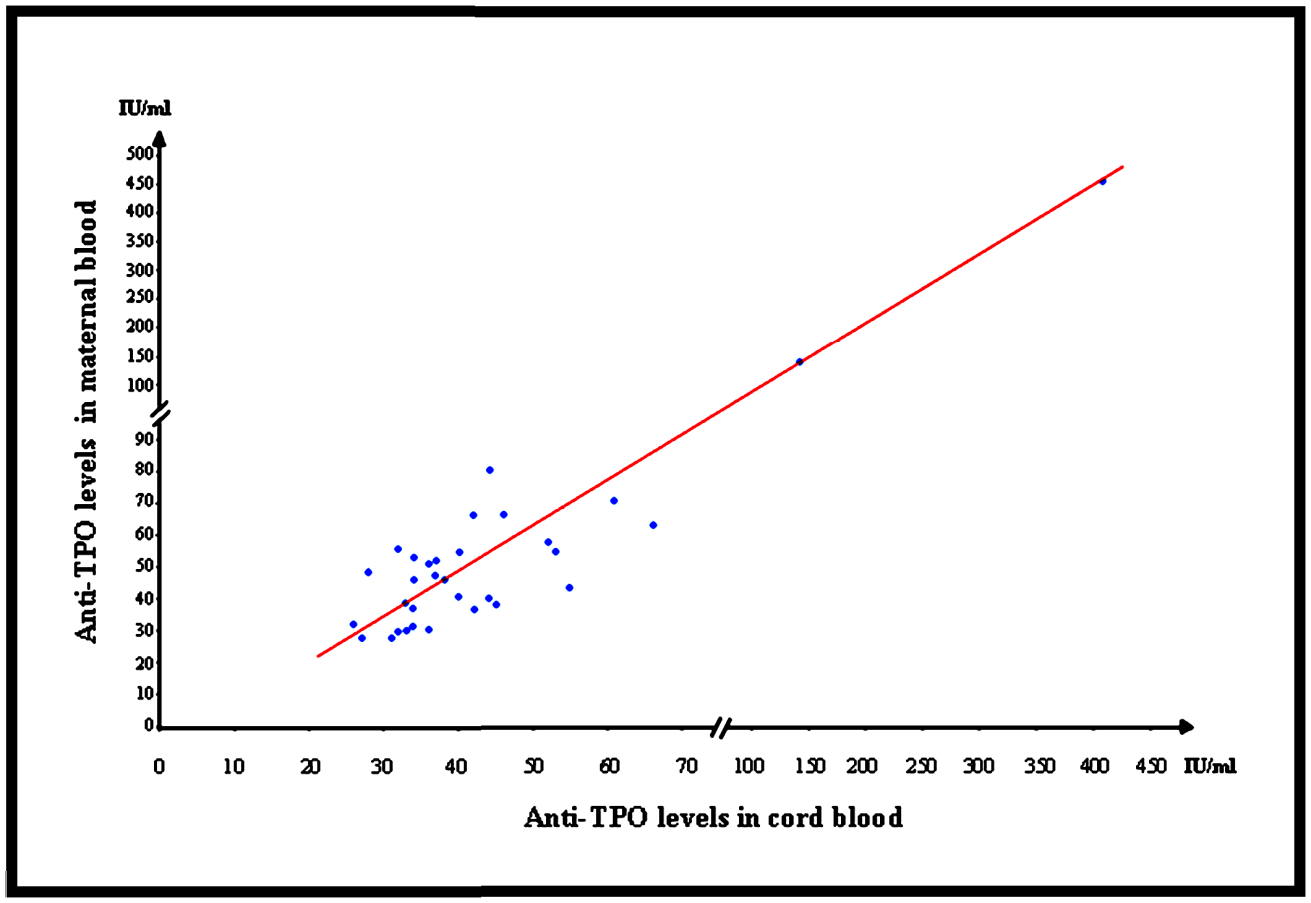
Correlation between anti-TPO levels in maternal blood and in cord blood at delivery.

The median anti-TPO level in maternal blood was 47 UI/ml [28; 467] and, in cord blood 38 UI/ml [26; 417]. There was no significative difference between the levels of anti-TPO in the maternal blood and the fetus in cord blood. We investigated the correlation between the anti-TPO levels in maternal blood and in cord blood and found a strong correlation with a correlation coefficient of 0.98 and a p-value<0.001 (Figure 2).

**Figure 3:**
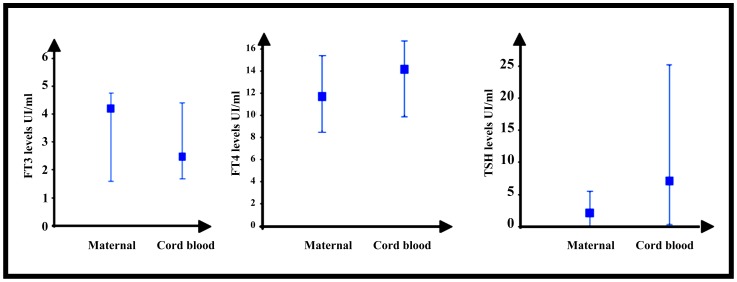
Median levels of FT3, FT4 and TSH between maternal and cord blood at delivery.

The FT3, FT4, and TSH median levels were respectively 4.1 UI/ml [3.3; 4.8], 12.1 UI/ml [9.5; 15.5], and 1.39 UI/ml [0.59; 2.99] in maternal blood at the third trimester, 4.2 UI/ml [1.5; 4.8], 11.8 UI/ml [8.7; 15.2], and 2.47 UI/ml [0.22; 5.18] at delivery and 2.5 UI/ml [1.6; 4.5], 14.3 UI/ml [10.1; 16.7], and 7.4 UI/ml [0.5; 24.94] in cord blood (Figure 3).

**Figure 4:**
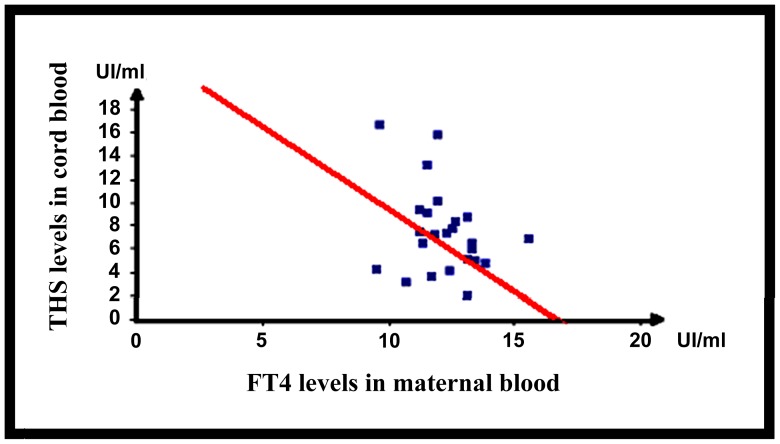
Correlation between FT4 levels in maternal blood and TSH levels in cord blood at delivery.

We found a correlation between the FT3 and FT4 levels in maternal blood at delivery and cord blood with a correlation coefficient respectively of 0.4 and 0.4 with a p-values = 0.02 and 0.01. In contrary, for TSH, there was no correlation between levels in maternal blood and cord blood with a correlation coefficient of 0.32 and a p-value = 0.11. Finally, we searched for a correlation between the FT4 levels in maternal blood at third trimester and TSH levels in cord blood. We found no correlation with a coefficient of 0.32 and a p-value = 0.10 in the population of anti-TPO positive mothers (n = 26) but a strong correlation with a coefficient of −0.4 with a p-value = 0.006 in the initial population of the study (n = 36) (Figure 4).

## Discussion

The main finding of this study is the first demonstration of the free passage through the placental barrier of anti-TPO from the mother to the fetus at the moment of childbirth with a strong correlation (p-value<0.001) and a “quasi linear” correlation coefficient of 0.98. These findings can not be reproduced during pregnancy for obvious ethical reasons but the characteristics of the placental barrier only slightly change after the first trimester of pregnancy and we can easily extrapolate these data all along pregnancy.

Hypothyroidism during pregnancy increases the risk to develop vasculo-placental complications such as gravidic hypertension (7.7%), pre-eclampsia (15.4%), preterm, abruption of placenta (3.8%), post partum haemorrhage (7.7%) and, post partum thyroiditis (19.2%) [10]. The effect on the fetus involving low-birth weight (15.4%) and fetal distress had been recognised [12], [22]. However, the association between the presence of anti-TPO, and such foeto-maternal complications has been observed [13] and many authors in literature believe that anti-TPO could play a role in the occurrence of these pathologies [15]. But so far, the pathogenicity of anti-TPO in pregnancy has not been clearly demonstrated. On the model of the demonstration of pathogenicity of antiphospholid antibodies [19], [20], a study on pregnant mice showed after passive immunization with purified human IgG with anti-TPO activity [15] a lower fetal birth weight and placental weight. Immunohistochimic study showed deposits of IgG inside the wall of uterine vessels highlighting a possible vascular mechanism of action of anti-TPO. A second study conducted on pregnant mice after passive immunization with recombinant mouse TPO, concluded to an increased fetal abortion rate related to circulating anti-TPO [21].

The demonstration of the passage of anti-TPO through the placental barrier is interesting on two points: first, it opens the door to a direct action of the anti-TPO on fetus and second to a possible action on the fetal thyroid. In fact, thyroid hormones are essential for fetal cerebral development [23], [24]. The role of maternal thyroid status on the future neuropsychological development of the fetus is important at all stages of pregnancy [25]. The maternal hypothyroidism is potentially deleterious to the cerebral development of the fetus [26]. Thyroid autoimmunity is also noted to be associated with a significant increase in women with spontaneous miscarriages. Furthermore, although thyroid antibodies are decreased during pregnancy, thyroid function deteriorates into subclinical hypothyroidism in women with thyroid autoimmunity. Moreover, these women have TSH levels in the first trimester higher compared with cases of hypothyroidism without antibodies. This finding remains true throughout pregnancy until delivery with levels of FT4 30% lower compared with those of women who are antibody-negative [10]. Then, there could be an interest in screening for hypothyroidism around the pregnancy [27].

From 11 weeks of gestation, fetal thyroid can produce thyroid hormones [28] and TSH is detectable in fetal serum [29]. In second and third trimester, a materno-fetal gradient for thyroid hormone concentrations is found in literature with higher levels of FT4 and FT3 in maternal than in fetus serum, despite an increase in fetal FT4, and FT3. We found similar results for FT3 levels at delivery but not for FT4 levels (Figure 3). This could be partly explained by the small number of serum samples in our study and the fact that this gradient decreases while approaching the end of pregnancy when the fetal thyroid function evolves [30]. However, TSH levels still remain superior in cord blood than in maternal blood (Figure 3). This is due to the relative insensitivity of the pituitary gland of the fetus to the retro control of FT4 [31], [32] but maybe also to the less sensitivity of fetal thyroid gland even if there is also placental transfer of FT3 and FT4 from the mother to the fetus, especially during late pregnancy [33].

Finally, in the population with anti-TPO positive mothers, there was no correlation between FT4 levels in maternal blood at third trimester and TSH levels in cord blood probably due to lack of power since in the entire population, we found a strong correlation. Theses findings suggest for the first time that the thyroid balance in mothers during pregnancy and particularly during third trimester could presume of thyroid activity in their fetus at delivery.

## Conclusion

Many authors in literature believe that anti-TPO could play a role in the occurrence of foeto-maternal complications during pregnancy and therefore, there is an interest in screening for hypothyroidism around the pregnancy. We report the first demonstration of the free passage through the placental barrier of anti-TPO from the mother to the fetus at the moment of childbirth. These findings can probably extrapolate to the third trimester of pregnancy and open the door to a direct action of the anti-TPO on fetus and to a possible action on the fetal thyroid function.

## References

[1] Tunbridge WM, Evered DC, Hall R, Appleton D, Brewis M, et al (1977) The spectrum of thyroid disease in a community: the Whickham survey. Clin Endocrinol 7: 481–93. 10.1111/j.1365-2265.1977.tb01340.x 598014

[2] Azarian M, Oury JF, Vuillard E, Legac I, Polak M, et al.. (2004) Anomalies thyroïdiennes en début de grossesse : que faire? In Mises à jour en gynécologie obstétrique. Editor: Collège National des gynécologues et obstétriciens Français, Paris, France. Publisher: Vigot, Paris, France. Tome XXVIII.

[3] Brown RS (1996) Autoimmune thyroid disease in pregnant women and their offspring. Endocr Pract 2: 53–61. 15251562 10.4158/EP.2.1.53

[4] Nguyen PH (2004) Autoimmune thyroid disease and pregnancy. In eMedicine world medical library. Editors: Ronald L, Francisco T, Carle VS, Fedrick BG, Lee PS.

[5] Lazarus JH, Rokandi A (2000) Thyroid disease in relation to pregnancy. A decade of change. Clin Endocrinol 53: 265–78. 10.1046/j.1365-2265.2000.01087.x 10971442

[6] Girling JC (2006) Thyroid disorders in pregnancy. Curr Obstet Gynaecol 16: 47–53.

[7] Smyth PP, Wijeyaratne CN, Kaluarachi WN, Smith DF, Premawardhana LD, et al (2005) Sequential studies on thyroid antibodies during pregnancy. Thyroid 15: 474–7. 15929669 10.1089/thy.2005.15.474

[8] Harborne LR, Alexander CE, Thomson AJ, O’Relly DSJ, Greer IA (2005) Outcomes of pregnancy complicated by thyroid disease. Aust NZ J Obstet Gynaecol 45: 239–42. 10.1111/j.1479-828X.2005.00365.x 15904452

[9] Lejeune B, Grun JP, de Nayer P, Servais G, Glinoer D (1993) Antithyroid antibodies underlying thyroid abnormalities and miscarriage or pregnancy induced hypertension. Br J Obstet Gynaecol 100: 669–72. 8369252 10.1111/j.1471-0528.1993.tb14236.x

[10] Nor Azlin M, Bakin Y, Mustafa N, Wahab N, Johari M, et al (2010) Thyroid autoantibodies and associated complications during pregnancy. J Obstet Gynaecol 30: 675–8. 20925608 10.3109/01443615.2010.503908

[11] Glinoer D, Soto MF, Bourdoux P, Lejeune B, Delange F, et al (1991) Pregnancy in patients with mild thyroid abnormalities: maternal and neonatal repercussions. J Clin Endocrinol Metab 73: 421–7. 1906897 10.1210/jcem-73-2-421

[12] Poppe K, Glinoer D (2003) Thyroid autoimmunity and hypothyroid before and during pregnancy. Hum Reprod Update 9: 149–61. 12751777 10.1093/humupd/dmg012

[13] Yin Lau L, Hang Pong N, Kam Shing L, Wei Min L, Wai Sum O, et al (2009) Increased fetal abortion rate in autoimmune thyroid disease is related to circulating TPO autoantibodies in an autoimmune thyroiditis animal model. Fertil Steril 91: 2104–9. 18774556 10.1016/j.fertnstert.2008.07.1704

[14] Ghafoor F, Mansoor M, Malik T, Malik MS, Khan AU, et al (2006) Role of thyroid peroxidase antibodies in the outcome of pregnancy. J Coll Physicians Surg Pak 16: 468–71. 16827958

[15] Seror J, Gilbert D, Laquerrière A, Arnoult C, Verspyck E, et al.. (2005) Pathogenic role of human IgG anti-thyroperoxidase antibodies passive immunization on Balb/c mouse gestation. In Sixièmes JNGOF congrès, Paris, France.

[16] De Escobar GM, Obregon MJ, Del Rey FE (2004) Maternal thyroid hormones early in pregnancy and fetal brain development. Best Pract Res Clin Endocrinol Metab 18: 225–48. 15157838 10.1016/j.beem.2004.03.012

[17] Montoro MN (1997) Management of hypothyroidism during pregnancy. Clin Obstet Gynecol 40: 65–80. 9103950 10.1097/00003081-199703000-00008

[18] Luton D, Alberti C, Vuillard E, Ducarme G, Oury JF, et al (2011) Iodine deficiency in northern Paris area: impact on fetal thyroid mensuration. PLoS One 6: e14707. 21359193 10.1371/journal.pone.0014707 PMC3040245

[19] Blank M, Cohen J, Toder V, Shoenfeld Y (1991) Induction of anti-phospholipid syndrome in naive mice with mouse lupus monoclonal and human polyclonal anti-cardiolipin antibodies. Proc Natl Acad Sci 88: 3069–73. 2014226 10.1073/pnas.88.8.3069 PMC51386

[20] Ziporen L, Blank M, Shoenfeld Y (1997) Animal models for antiphospholipid syndrome in pregnancy. Rheum Dis Clin North Am 23: 99–117. 9031377 10.1016/s0889-857x(05)70317-3

[21] Lee YL, Ng HP, Lau KS, Liu WM, O WS, et al (2009) Increased fetal abortion rate in autoimmune thyroid disease is related to circulating TPO autoantibodies in an autoimmune thyroiditis animal model. Fertil Steril 91: 2104–9. 18774556 10.1016/j.fertnstert.2008.07.1704

[22] Männistö T, Vääräsmäki M, Pouta A, Hartikainen AL, Ruokonen A, et al (2009) Perinatal outcome of children born to mothers with thyroid dysfunction or antibodies: a prospective population-based cohort study. J Clin Endocrinol Metab 94: 772–9. 19106271 10.1210/jc.2008-1520

[23] Oken E, Braverman LE, Platek D, Mitchell ML, Lee SL, et al (2009) Neonatal thyroxine, maternal thyroid function, and child cognition. J Clin Endocrinol Metab 94: 497–503. 19033373 10.1210/jc.2008-0936 PMC2646520

[24] Williams GR (2008) Neurodevelopmental and neurophysiological actions of thyroid hormone. J Neuroendocrinol 20: 784–94. 18601701 10.1111/j.1365-2826.2008.01733.x

[25] Morreale de Escobar G, Obregon MJ, Escobar del Rey F (2004) Role of thyroid hormone during early brain development. Eur J Endocrinol 151: 25–37. 10.1530/eje.0.151u025 15554884

[26] Haddow J, Palomaki G, Allan W, Williams J, Knight G, et al (1999) Maternal thyroid deficiency during pregnancy and subsequent neuropsychological development of the child. N Engl J Med 341: 549–55. 10451459 10.1056/NEJM199908193410801

[27] De Groot L, Abalovich M, Alexander EK, Amino N, Barbour L, et al (2012) Management of thyroid dysfunction during pregnancy and postpartum : an Endocrine Society clinical practice guideline. J Clin Endocrinol Metab 97: 2543–65. 22869843 10.1210/jc.2011-2803

[28] Goodwin T, Montoro M, Mestman J (1992) Transient hyperthyroidism and hyperemesis gravidarium: clinical aspects. Am J Obstet Gynecol 176: 648–52. 10.1016/s0002-9378(11)91565-8 1382389

[29] Schlienger J (2001) Thyroïde et grossesse. Hypothyroïdie et grossesse. In La thyroïde, des concepts à la pratique clinique. 2^ème^ édition. Leclere J, Orgiazzi J, Rousset B, Schlienger J, Wemeau JL. Editor :Editions scientifiques et médicales Elsevier SAS, Paris, France. Publisher :Bialec, Nancy, France. Chapitre 93: 503–6.

[30] Elahi S, Laeeq F, Syed Z, Rizvi SMH, Hyde RSW (2005) Serum thyroxine and thyroid stimulating hormone levels in maternal circulation and cord blood at the time of delivery. Pak J Med Sci 21: 325–30.

[31] Thorpe-Beeston JG, Nicolaides KH, Felton CV, Butler J, McGregor AM (1991) Maturation of the secretion of thyroid hormone and thyroid-stimulating hormone in the fetus. N Engl J Med 324: 532–36. 1899469 10.1056/NEJM199102213240805

[32] Kuppens S, Kooistra L, Wijnen HA, Vader HL, Hasaart T, et al (2011) Neonatal thyroid screening results are related to gestational maternal thyroid function. Clin Endocrinol 75: 382–87. 10.1111/j.1365-2265.2011.04083.x 21521349

[33] Vulsha T, Gons MH, de Vijlder JJM (1989) Maternal-fetal transfer of thyroxine in congenital hypothyroidism due to a total organification defect or thyroid agenesis. N Engl J Med 321: 13–6. 2733742 10.1056/NEJM198907063210103

